# AI-2 of *Aggregatibacter actinomycetemcomitans* inhibits *Candida albicans* biofilm formation

**DOI:** 10.3389/fcimb.2014.00094

**Published:** 2014-07-21

**Authors:** Endang W. Bachtiar, Boy M. Bachtiar, Lucja M. Jarosz, Lisa R. Amir, Hari Sunarto, Hadas Ganin, Michael M. Meijler, Bastiaan P. Krom

**Affiliations:** ^1^Department of Oral Biology, Faculty of Dentistry, Universitas IndonesiaJakarta, Indonesia; ^2^Department of Biomedical Engineering, The W.J. Kolff Institute, University Medical Center Groningen and University of GroningenGroningen, Netherlands; ^3^Department of Periodontology, Faculty of Dentistry, Universitas IndonesiaJakarta, Indonesia; ^4^Department of Chemistry, Ben-Gurion University of the NegevBe'er-Sheva, Israel; ^5^Department of Preventive Dentistry, Academic Centre for Dentistry Amsterdam (ACTA), University of Amsterdam and Free University AmsterdamAmsterdam, Netherlands

**Keywords:** oral microbiology, interspecies interaction, quorum sensing

## Abstract

*Aggregatibacter actinomycetemcomitans*, a Gram-negative bacterium, and *Candida albicans*, a polymorphic fungus, are both commensals of the oral cavity but both are opportunistic pathogens that can cause oral diseases. *A. actinomycetemcomitans* produces a quorum-sensing molecule called autoinducer-2 (AI-2), synthesized by LuxS, that plays an important role in expression of virulence factors, in intra- but also in interspecies communication. The aim of this study was to investigate the role of AI-2 based signaling in the interactions between *C. albicans* and *A. actinomycetemcomitans*. *A. actinomycetemcomitans* adhered to *C. albicans* and inhibited biofilm formation by means of a molecule that was secreted during growth. *C. albicans* biofilm formation increased significantly when co-cultured with *A. actinomycetemcomitans luxS*, lacking AI-2 production. Addition of wild-type-derived spent medium or synthetic AI-2 to spent medium of the *luxS* strain, restored inhibition of *C. albicans* biofilm formation to wild-type levels. Addition of synthetic AI-2 significantly inhibited hypha formation of *C. albicans* possibly explaining the inhibition of biofilm formation. AI-2 of *A. actinomycetemcomitans* is synthesized by LuxS, accumulates during growth and inhibits *C. albicans* hypha- and biofilm formation. Identifying the molecular mechanisms underlying the interaction between bacteria and fungi may provide important insight into the balance within complex oral microbial communities.

## Introduction

*Aggregatibacter actinomycetemcomitans* is a non-motile, Gram-negative coccobacillus, which can be found as a commensal in the oral cavity. In addition, it is also the principal cause of aggressive periodontal disease (Saarela et al., 1999). *A. actinomycetemcomitans* uses chemical signals to sense cell density and alter expression of virulence factors (Novak et al., 2010). This microbial communication is known as quorum sensing (QS) and the only identified cell-cell signaling molecule in *A. actinomycetemcomitans* this far is autoinducer 2 (AI-2). AI-2, which has been proposed to be a general interspecies, concentration dependent signal, is synthesized by LuxS as a precursor, 4,5,-dihydroxy-2,3-pentanedione (DPD), followed by secretion into the medium where it spontaneously undergoes cyclization into AI-2 which accumulates. Interestingly, DPD can thus be converted into several structures that can be recognized by different species (Miller et al., 2004). For instance, *Vibrio harveyi* produces an unusual furanosyl borate diester ((3aS,6S,6aR)-2,2,6,6a-tetrahydroxy-3a-methyltetrahydrofuro[3,2-d][1,3,2] dioxaborol-2-uide), while *Salmonella* Typhimurium recognizes (2R,4S)-2-methyl-2,3,3,4-tetrahydroxytetrahydrofuran.

In addition, the polymorphic fungus *Candida albicans* is one of the most commonly isolated fungi from the oral cavity (Cannon and Chaffin, 1999). In healthy individuals, *C. albicans* grows as a commensal, mostly in the unicellular yeast morphology, but in immune-compromised individuals this species is capable of producing multicellular filamentous forms, a pathogenic morphology (Odds, 1988). The morphological transition from the yeast-to-hyphal mode of growth is influenced by many factors, including pH, nutrient availability, temperature and the presence of QS molecules, that stimulate or repress filamentation as a function of cell density (Hornby et al., 2001; Hazan et al., 2002). Recently, the interaction of bacteria with *C. albicans* through QS molecules has received increasing attention (Shirtliff et al., 2009). In the oral cavity two Gram-positive bacteria have been shown to affect *C. albicans* biofilm formation through QS molecules. *Streptococcus mutans* was shown to inhibit hyphal formation through the QS molecule competence-stimulating peptide (CSP) (Jarosz et al., 2009) and the fatty acid signaling molecule trans-2-decenoic acid (Vilchez et al., 2010). In addition, *S. gordonii* was shown to secrete AI-2, which was shown to repress *C. albicans* QS by inhibiting the action of farnesol (Bamford et al., 2009) upon contact. In the healthy oral cavity a multitude of bacterial species co-exist with *C. albicans*, both Gram-negative and Gram-positive (Zaura et al., 2009). In contrast to the limited information on the effect of Gram-positive oral bacteria on *C. albicans*, no information is available on the effect of QS molecules of Gram-negative oral bacteria. Therefore, in the present study, it was our aim to investigate the effect of AI-2 produced by the Gram-negative oral bacterium *A. actinomycetemcomitans* on *C. albicans*.

## Materials and methods

### Microbial strains and growth conditions

*A. actinomycetemcomitans* spp. were routinely cultured for 18 h at 37°C under microaerobic conditions (10% CO_2_) on trypticase soy agar or broth containing 0.6% yeast extract (TSB-YE/Difco). When appropriate, standardized cell suspensions were prepared with a density of 2.1 × 10^8^ CFU mL^−1^ as determined with serial dilution plating and determination of the optical density measured at 655 nm (OD_655_). *Escherichia coli* was routinely grown in Luria-Bertani broth at 37°C with constant aeration. For solid medium, 15 g agar per liter was added to the liquid medium. When required, ampicillin (100 μg mL^−1^) or kanamycin (30 μg mL^−1^) was added to the medium. *C. albicans* strain ATCC 10231 or SC5314 were taken from stock cultures frozen in 15% glycerol at −80°C and sub-cultured twice onto yeast peptone agar plates with 2% glucose (YPD) or when indicated in yeast nitrogen base medium pH 7, supplemented with 50 mM glucose (YNB).

### Adhesion of *A. actinomycetemcomitans* to *C. albicans*

Adhesion of *A. actinomycetemcomitans* Y4 to *C. albicans* strain SC5314 was studied using a Bioflux 1000Z setup. Briefly, *C. albicans* was seeded into a 48-well microfluidics plate (Fluxion Biosciences) at an initial optical density measured at 600 nm of 0.2 (OD_600_ = 0.2). After 30 min adhesion to the bottom plate at 37°C, flow with prewarmed YNB was started at 0.5 dyne cm^−2^ for 4 h. A bacterial suspension at an initial OD_600_ = 0.2 in phosphate buffered saline (PBS; 10 mM potassium phosphate, 0.15 M sodium chloride, pH 7) containing 0.2 μL mL^−1^ Syto9 and 0.2 μL mL^−1^ propidium iodide (*Bac*light, Invitrogen) was flowed through the microfluidics channels at 0.5 dyne cm^−2^ and images were captured every 30 s using the appropriate filter settings. Images were translated to AVI-movies using ImageJ (1.46r).

### Construction of an *A. actinomycetemcomitans luxs* mutant

*A. actinomycetemcomitans* (serotype b) was isolated in our periodontal clinic (Universitas Indonesia, Jakarta) from periodontitis patients, with their consent. The isolated bacteria were identified by means of Gram staining, characteristic star-positive colonies on agar plates and tight adherence to surfaces when grown in broth (Slots et al., 1982). Positive clones were confirmed and serotyped using PCR (Suzuki et al., 2001). A single isolate, *A. actinomycetemcomitans* UI-09, was selected as wild-type strain for all other experiments. In addition, the commonly used *A. actinomycetemcomitans* Y4 was used as a reference in certain experiments and showed similar results compared to *A. actinomycetemcomitans* UI-09.

All primers for plasmid construction were designed using *A. actinomycetemcomitans* database (http://www.oralgen.org, ID for *luxS* in *A. actinomycetemcomitans* HK 1651 is AA00516). In order to create a *luxS* defective mutant, a suicide vector was constructed using the neighbor-joining technique as previously described for *Campylobacter jejuni* (Bachtiar et al., 2007). Firstly, a 229-bp DNA fragment containing part of the upstream sequence adjacent to *luxS* was PCR-amplified using the primer *EcoR*I-L1 (ACGAATTCAATCCACCGCACTT, forward) and primer *BamH*I-L2 (TCGGATCCAAGTTTTCTTGTTAGG, reverse). The PCR product was cloned into pBluescript in the forward direction via the *EcoR*I and *BamH*I sites, and confirmed by restriction analysis. The construct (pBl-L1) was subsequently introduced into *E. coli* JM 107. Positive clones were selected on LB agar supplemented with ampicillin, X-Gal and IPTG. Secondly, a 409-bp DNA fragment containing the downstream flanking region of *luxS* was amplified using primers *BamH*1-L3 (CATGGATCCGAAGAAGCACATCAA, forward) and *Xba*I-L4 (ATCTAGAGCAAGTTGCTCGTAA, reverse). The amplified fragment was inserted into pBl-L1 between *Bam*HI and *Xba*I sites. The resulted intermediate plasmid (pBl-L2) was cut at the unique *BamH*I site and a 1.4-kbp fragment containing a kanamycin cassette (obtained from vector pMW2 digested with *BamH*I) was ligated into the plasmid to obtain the suicide plasmid, pBLkm^r^.

### Natural transformation

A biphasic system for *A. actinomycetemcomitans* transformation was performed as described previously (Wang et al., 2002). Transformation was done by incubating a suspension of 10^8^ CFU mL^−1^ of *A. actinomycetemcomitans* at 37°C under microaerobic conditions for 3 h. Subsequently, 10 μg of the suicide vector was added and cells were incubated for 3 h at 37°C. Cells were then harvested and plated on medium supplemented with kanamycin and incubated at 37°C, under microaerobic conditions for 2 days to select for transformants. Homologous recombination and disruption of *luxS* was confirmed using PCR analysis with primers flanking the target site (*EcoR*I-L1 and *Xba*I-L4).

### Spent medium preparation

Spent medium of *A. actinomycetemcomitans* cultures was prepared as described previously (Jarosz et al., 2009). Protein concentration in the spent medium was measured using the Bradford method. Spent medium was diluted in PBS to yield 10 and 100 μg mL^−1^ concentrations and used immediately or stored for short periods of time at −20°C. The pH of the spent medium was adjusted to pH 7.

### Synthesis of (S)-4,5,-dihydroxy-2,3-pentanedione (DPD)

DPD was synthesized following a procedure published by Ganin et al. (2009). Lyophilized DPD was dissolved in DMSO at 33 mM stock concentrations and stored at −20°C until required. Synthetic DPD concentrations used in the described experiments are assumed to be physiologically relevant as they are in line with concentrations of AI-2 reported to be produced in saliva-fed natural oral biofilms (Rickard et al., 2008).

### Biofilm formation of *C. albicans* and *A. actinomycetemcomitans*

Quantification of *A. actinomycetemcomitans* biofilms was achieved by staining with Crystal Violet (CV). *A. actinomycetemcomitans* strains (5 μL) were used to inoculated wells of 96-well (flat-bottom) cell culture plates (Sumitomo Bakelite Co., Ltd, Tokyo, Japan) containing 95 μL TSB-YE in each well. After, 18 h of incubation under microaerobic condition, the culture medium containing planktonic cells was removed and the wells were carefully washed with 200 μL of distilled water. Adherent bacteria were stained with 50 μL of 0.1% CV for 15 min at room temperature. After rinsing twice with 200 μL of distilled water, CV bound to the biofilm was extracted with 200 μL of 99% ethanol for 20 min and quantified by measuring the absorbance at 655 nm with a microplate reader (Model 3550, Bio-Rad Laboratories, Hercules, CA, USA).

Biofilm formation of *C. albicans* was induced in YNB as previously reported (Krom et al., 2007). Mixed species biofilms of *C. albicans* (2 × 10^6^ CFU mL^−1^) and *A. actinomycetemcomitans* (2.1 × 10^7^ CFU mL^−1^) were grown in medium containing 70% YNB and 30% TSB-YE (vol/vol). Where indicated, the 30% fresh TSB-YE fraction was replaced by spent medium as described previously (Jarosz et al., 2009). In addition, when indicated, sterile spent medium from the *A. actinomycetemcomitans* was added to YNB at 1, 10, and 100 μg mL^−1^ protein concentration. After 24 and 48 h of growth, biofilm formation on the well of microtiter plates were washed once with PBS and metabolic activity of the biofilms was quantified using [3-(4,5-dimethyl-2-thiazolyl)-2,5-diphenyl-2H-tetrazolium bromide] (MTT) as described previously (Krom et al., 2007). Since *C. albicans* rapidly metabolized MTT, in contrast to *A. actinomycetemcomitans* (Supplementary Figure S1) this assay could be used to determine *C. albicans* biofilm formation even in co-cultures with *A. actinomycetemcomitans*. All assays were carried out on at least two separate occasions with at least duplicate determinations on each occasion. Microtiter wells containing only YNB broth but no cells were used as negative controls. To mimic the conditions in the oral cavity initial experiments were also performed in the presence of pooled sterilized human saliva, however no differences were observed relative to experiments without saliva. Therefore, all other experiments were performed without saliva.

### Inhibition of hypha formation

Hypha formation was assayed as described previously (Jarosz et al., 2009) using *C. albicans* strain SC5314. The morphology of cells was analyzed using a 20x objective on an inverted microscope (Olympus, Tokyo, Japan).

### Statistical analyses

Differences between means were analyzed for statistical significance using two-tailed Student's *t*-tests. Differences were considered significant when *p* ≤ 0.05 level. For multiple comparisons, a One-Way ANOVA followed by a TUKEY HSD test was performed (http://vassarstats.net/anova1u.html).

## Results

### Adhesion of *A. actinomycetemcomitans* to *C. albicans*

**Figure 1:**
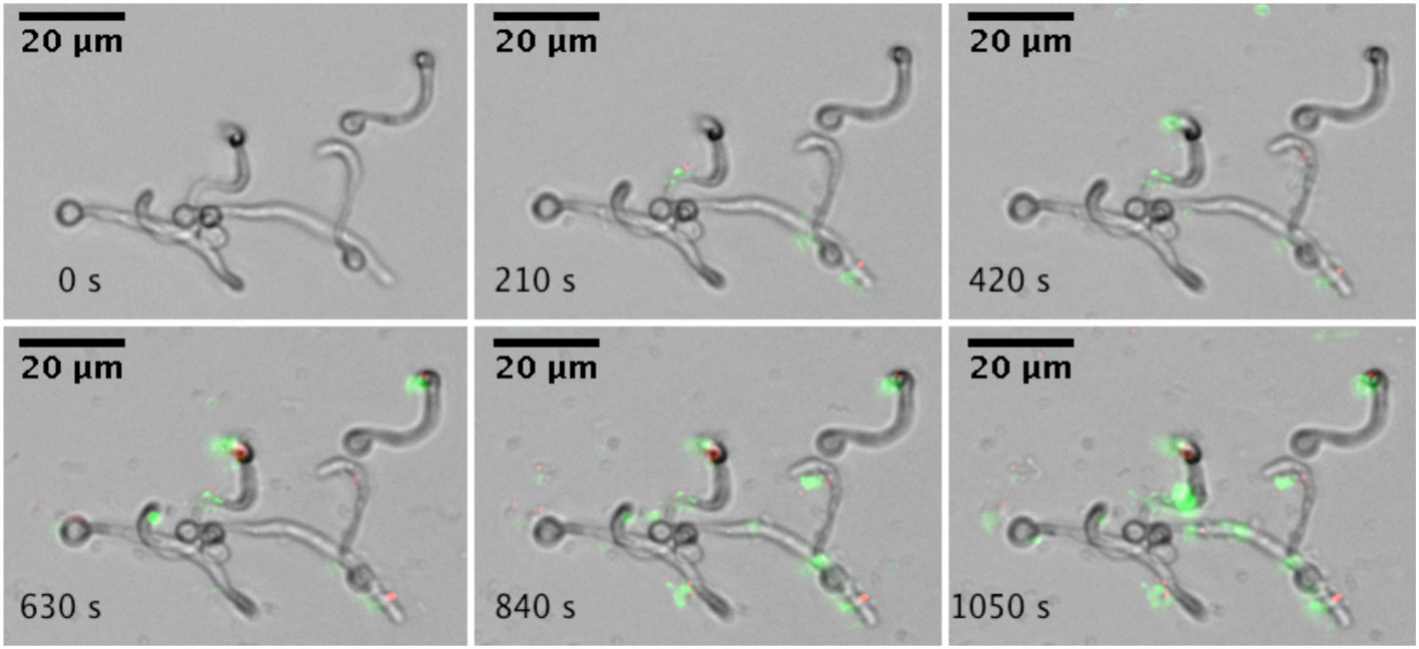
**Real-time microscopic analysis of adhesion of *A. actinomycetemcomitans* to *C. albicans* SC5314**. Hyphae of *C. albicans* SC5314 were allowed to form on the bottom of a microfluidics plate. Bacteria were stained with Syto9 and PI and allowed to adhere to *C. albicans* SC5314 while flowing at 0.5 dyne cm^−2^. Images were captured every 30 s for a total of 10 min (montage shows images every 210 s). Images were edited for brightness and contrast.

Adhesion to surfaces is a critical initial step in microbial biofilm formation. Using a microfluidics setup, adhesion under flow conditions were studied. Single *A. actinomycetemcomitans* Y4 cells adhere to hyphae and yeast cells of *C. albicans* SC5314, as illustrated by the increased green fluorescent spots associated with *C. albicans* (Figure 1). In addition, adhesion of *A. actinomycetemcomitans* to the glass surface could be observed.

### Effects of *luxs* deletion on biofilm formation of *A. actinomycetemcomitans*

**Figure 2:**
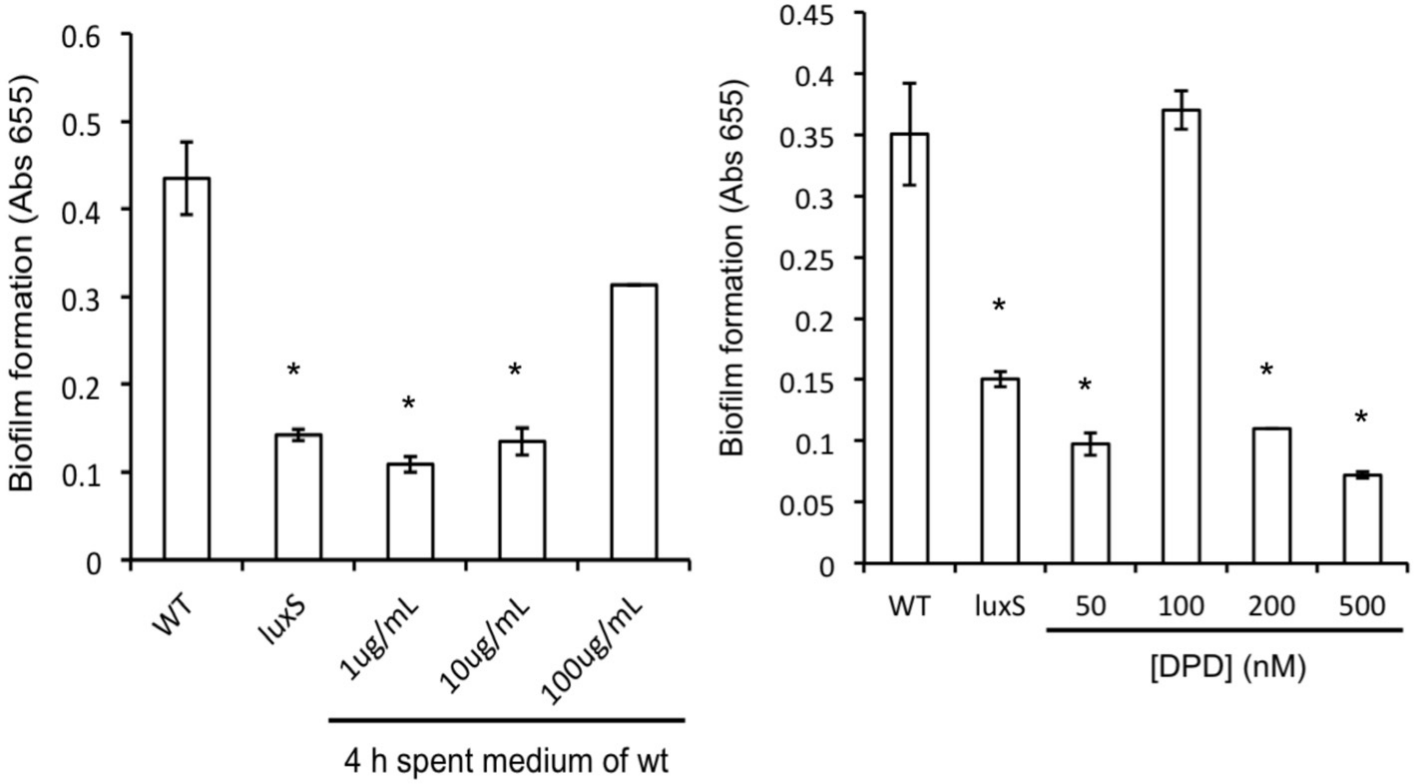
**AI-2 plays a role in biofilm formation of *A. actinomyc-etemcomitans***. Biofilm formation of *A. actinomycetemcomitans luxS* is restored to near wild-type levels by the addition of sterile spent medium **(left panel)** as well as by the addition of 100 nM synthetic DPD **(right panel)**. ^*^Indicates significantly different from control.

*A. actinomycetemcomitans luxS* formed significantly less biofilm compared to the wild type strain (Figure 2) but growth rate in planktonic cultures was not affected, in line with previous reports (Novak et al., 2010). Spent medium of a 4 h-old culture of the wild type strain was able to rescue biofilm formation of the *luxS* strain at 100 μg protein mL^−1^ (Figure 2, left panel). Medium of 6 h-old cultures also rescued the phenotype to a similar, but not medium of 8 and 24 h-old cultures. Addition of synthetic DPD rescued this phenotype specifically at 100 nM DPD, but not at lower or higher concentrations (Figure 2, right panel).

### Role of AI-2 on mixed species biofilms of *A. actinomycetemcomitans* and *C. albicans*

**Figure 3:**
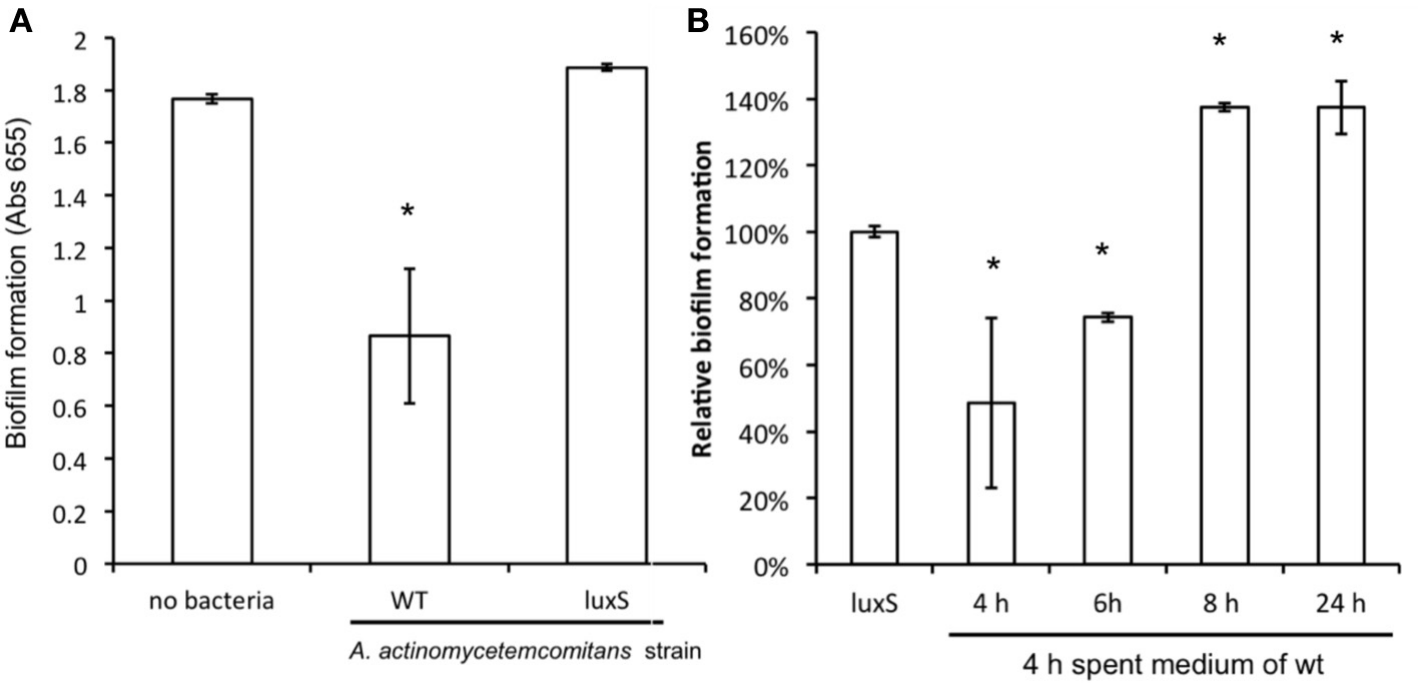
**The effect of co-culture with *A. actinomycetemcomitans* on *C. albicans* ATCC 10231 biofilm formation. (A)** *C. albicans* biofilms were grown without any bacteria or with *A. actinomycetemcomitans* wild-type or *luxS* mutant. Biofilm formation, quantified using the MTT assay, was measured after 24 h of growth. Data represent the mean and standard deviations of six biofilms grown on two separate occasions. **(B)** The effect of spent medium on co-cultures between *C. albicans* and *A. actinomycetemcomitans luxS*. Spent medium of *A. actinomycetemcomitans* wild-type cultures grown for different times was added to the mixed species culture of *C. albicans* and *A. actinomycetemcomitans luxS*. Biofilm formation was quantified after 24 h of growth using the MTT assay. The relative biofilm formation compared to *C. albicans* + *A. actinomycetemcomitans luxS* without any spent medium was calculated. Data represent the mean and standard deviations of six biofilms grown on two separate occasions. ^*^Indicates significantly different from control.

Compared to mono-species biofilm formation of *C. albicans*, mixed species biofilms of *C. albicans* with *A. actinomycetemcomitans* resulted in a reduction of more than 50% in metabolic activity after 24 h of culturing (Figure 3A). A similar decrease in biofilm formation was observed after 48 h of culturing (not shown). Mixed species biofilms of *C. albicans* with *A. actinomycetemcomitans luxS* had no significant effect compared to mono-species biofilms.

### Effect of secreted factors on *C. albicans* biofilm formation

To further determine whether the inhibition effect was modulated by secreted compounds of the bacteria, the spent medium of the wild type *A. actinomycetemcomitans* was used as a source of secreted molecules to complement *A. actinomycetemcomitans luxS*. When spent medium from 4 and 6 h-old wild-type cultures was added, the *A. actinomycetemcomitans luxS* mutant was able to inhibit *C. albicans* biofilm formation, but this inhibition was not observed for spent medium derived from 8 and 24-h old cultures (Figure 3B). Conversely, when spent medium of *A. actinomycetemcomitans luxS* was added to mixed species biofilms of *C. albicans* and *A. actinomycetemcomitans luxS*, no significant inhibitions of biofilm formation was observed (Supplementary Figure S2).

### Effect of synthetic DPD on *C. albicans* biofilm growth and hypha formation

**Figure 4:**
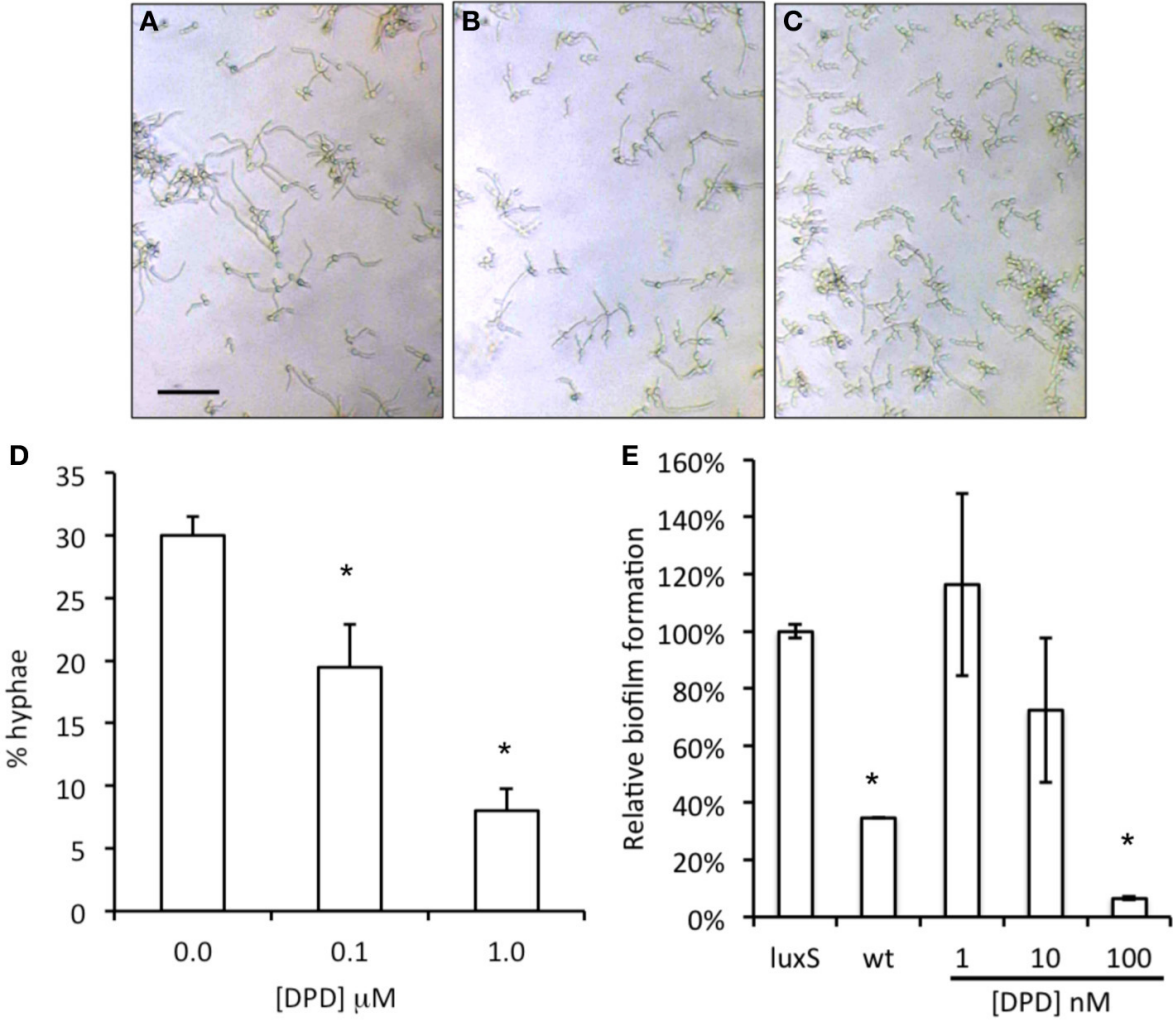
**Effect of synthetic DPD on *C. albicans* SC5134**. Hypha-formation was induced by switching fresh cultures to 37°C for 3–4 h. Representative microscopic images show a decreased hypha formation (**A** = control, **B** = 0.1 μM DPD, **C** = 1.0 μM DPD; bar represent 40 μm for all images). Hyphae and yeast morphologies were counted and plotted as % of all cells **(D)**. The results represent the mean of two independent experiments, each consisting of at least 100 cells per sample. Synthetic DPD added to spent medium of *A. actinomycetemcomitans luxS* inhibits *C. albicans* SC5314 biofilm formation **(E)**. ^*^Indicates significantly different from control.

When synthetic DPD was added to spent medium of *A. actinomycetemcomitans luxS* during *C. albicans* biofilm growth, a concentration dependent inhibition of *C. albicans* biofilm formation was observed with the maximum inhibition reached at 100 nM DPD (Figure 4, right panel). Hypha formation is a key process in biofilm formation. Synthetic DPD was added to *C. albicans*‘ under hypha inducing conditions. DPD inhibited hypha formation by 30 and 70% at 100 nM and 1 μM, respectively (Figure 4, left panel).

### *A. actinomycetemcomitans* spent medium disrupts established *C. albicans* biofilm

**Figure 5:**
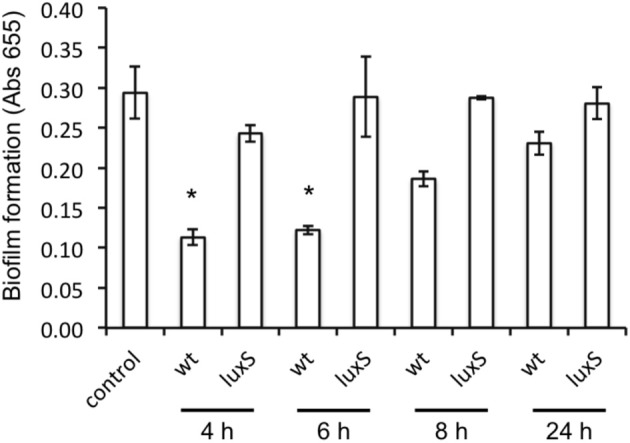
**Effect of spent medium of *A. actinomycetemcomitans* wt and *luxS* on preformed *C. albicans* ATCC 10231 biofilms**. Biofilms of *C. albicans* were grown for 24 h after which they were exposed to spent medium of *A. actinomycetemcomitans* cultures of increasing age for an additional 24 h. Total amount of biofilm was determined using the MTT assay and results depict the averages of a total of 4 wells in 2 independent experiments. ^*^Indicates significantly different from control as determined using a One-Way ANOVA followed by a TUKEY HSD test.

To test the ability of *A. actinomycetemcomitans* to disrupt *C. albicans* biofilms, we challenged established biofilms (24 h old) of *C. albicans* with spent medium from *A*. *actinomycetemcomitans* WT and *luxS* cultures of increasing age. A culture age dependent decrease in viability was observed when *C. albicans* biofilms were exposed to spent medium of cultures of the WT strain, but not to spent medium derived from the same aged cultures of the *luxS* strain (Figure 5).

## Discussion

*A. actinomycetemcomitans* is related to severe periodontitis. In addition, several studies have reported on the isolation of a large number *Candida* spp., from periodontal pockets of patients with periodontitis (Jarvensivu et al., 2004; Urzua et al., 2008). Recently, co-isolation of *Candida* spp. and *A. actinomycetemcomitans* was correlated with the occurrence of severe periodontitis (Brusca et al., 2010). However, due to limited information it is currently unclear if *C. albicans* participates in the etiology of any kind of periodontitis. Biofilms are the most common mode of growth of *Candida spp*., as observed *in vivo*. Therefore, interspecies interactions occur preferentially in mixed species biofilms such as those found in the periodontal pocket. A better fundamental understanding of interspecies interaction in the oral cavity is therefore of great relevance. Here we show for the first time that a commonly isolated periodontal pathogen *A. actinomycetemcomitans* adheres to *C. albicans* under flow condition (Figure 1). Physical interactions, such as adhesion are initial and probably critical stages in microbial biofilm formation. Following adhesion growth and accumulation results in interspecies chemical interactions, amongst others mediated by quorum sensing (Jarosz and Krom, 2011).

AI-2 activity has been discovered in spent culture supernatants of many oral bacteria (Fong et al., 2001; Blehert et al., 2003; Wen and Burne, 2004). However, the function of AI-2 as a general bacterial signaling molecule is an issue that is yet to be resolved. Several studies have suggested that AI-2 is involved in biofilm formation (Chung et al., 2001; Yoshida et al., 2005). On the other hand, it has also been suggested that the main function of the LuxS enzyme is in the regulation of metabolic processes (Winzer et al., 2002). Inactivation of *luxS* in *A. actinomycetemcomitans* does not affect growth rates but does result in phenotypic alterations, including biofilm formation and reduced colonization in various experimental infection models (Hardie and Heurlier, 2008). Our data demonstrated that biofilm formation of the *A. actinomycetemcomitans luxS* strain was decreased as compared with its parent strain. Complementation of the *luxS* deletion by both spent medium of wild-type *A. actinomycetemcomitans* as well as by addition of synthetic DPD would indicate that the *luxS* mutant phenotype is due to absence of the quorum sensing molecule and not related to any metabolic defect. It should however be noted that it is not straightforward to uncouple signaling and metabolic functions of LuxS (Redanz et al., 2012). The remarkable concentration dependent complementation of the *luxS* mutant by DPD—reaching a maximum at 100 nM, followed by a sharp decrease at higher concentrations - is interesting and currently we are unable to explain this specific behavior. It is however important to notice that such a behavior has been observed in another study. Rickard and coworkers described a similar sharp concentration optimum for synthetic AI-2 in a mixed-species biofilm model consisting of *Actinomyces naeslundii* and *Streptococcus oralis* be it at approximately 100-fold lower concentration (Rickard et al., 2006). Spent medium of 8 h cultures and older no longer inhibited *C. albicans* biofilm formation (see Figure 5). This could be related to decreased AI-2 presence at later stages of growth which is in line with the growth-phase specific production of AI-2, higher in the first 6 h of growth compared to later stages, observed in several oral species (Fong et al., 2001; Blehert et al., 2003; Wen and Burne, 2004).

Bamford et al. showed that *S. gordonii* increases *C. albicans* biofilm formation in a LuxS dependent fashion, however, no effect of synthetic DPD on *C. albicans* hypha formation was observed (Bamford et al., 2009). In contrast, we observed that the exogenous addition of AI-2/DPD to the growth medium restored the *luxS* phenotype in a dose-dependent manner and that addition of synthetic DPD inhibited hypha formation of *C. albicans*. This effect was only seen in medium consisting of 70% YNB/30% TSB, and not in YNB alone. In contrast to Bamford we did not use saliva in the germ-tube assay. Both minimal medium and saliva are strong inducers of hypha formation and this might explain the observed different response of *C. albicans* to synthetic DPD. Additionally, use of different *C. albicans* strains in the present study compared to Bamford and coworkers could account for the observed differences in response. Alternatively, the different response under different hypha inducing conditions could indicate a difference in the chemical structure of AI-2 produced by *S. gordonii* or *A. actinomycetemcomitans*, and intriguingly that *C. albicans* is able to recognize this chemical difference. Detailed chemical characterization of the AI-2 for both species should be performed to further support this statement.

Communication within oral microorganisms involves several classes of signal molecules; autoinducing peptides synthesized by Gram-positive bacteria, such as competence-stimulating peptide of *S. mutans* (Jarosz et al., 2009), fatty acid signaling molecules such as farnesol and trans-2-decenoic acid (SDSF) (Vilchez et al., 2010) and AI-2 as a proposed universal signal produced by both Gram-positive and Gram-negative bacteria (Federle and Bassler, 2003). The different responses of *C. albicans* to AI-2, depending on its origin, is intriguing and could illustrate that the already complicated interspecies chemical communication might be even more complicated, but a more extensive study is needed to provide solid evidence for such a tempting, but speculative conclusion.

Oral biofilms are very complex multi-species communities in which we assume that interspecies interaction plays a role in establishing and maintaining a balance. A recent study on bacterial and yeast colonization in a group of mucositis patients showed a complete absence of *A. actinomycetemcomitans* and a more than average presence of *C. albicans* (Laheij et al., 2012). As hypha formation is a pivotal step in *C. albicans* biofilm formation (Krueger et al., 2004) and adhesion, the LuxS mediated interaction with *A. actinomycetemcomitans* would decrease fungal biofilm formation. Additionally, the function of LuxS is related to the regulation of bacteria virulence factors, including cytolethal distending toxin (CDT), as reported in *Campylobacter jejuni* (Jeon et al., 2005). For *A. actinomycetemcomitans* it has been shown that CDT (CDTB) is toxic to the yeast *Saccharomyces cereviseae* (Matangkasombut et al., 2010). There are therefore potentially multiple systems, one based on a secreted QS molecule and a second on a toxin, involved in the observed negative interactions between *A. actinomycetemcomitans* and *C. albicans*. A preliminary study indicated that spent medium of wild-type *A. actinomycetemcomitans* has no toxic effects on *C. albicans* (Supplementary Figure S3). Similar dual mechanisms of inhibition have been observed between *Staphylococcus aureus* and *Pseudomonas aeruginosa* (Li and Krom, unpublished data). It is tempting to hypothesize that the QS molecule is sensed as a defense mechanism to protect against the upcoming battle with a toxin-producing competitor (Jarosz et al., 2011). Additional studies are required to further elucidate the molecular and biochemical mechanisms involved in the interspecies interaction between *A. actinomycetemcomitans* and *C. albicans*.

### Conflict of interest statement

The authors declare that the research was conducted in the absence of any commercial or financial relationships that could be construed as a potential conflict of interest.

## Supplementary material

Supplementary Figure S1 **Comparison of MTT and XTT reduction by *C. albicans* SC5314 and *A. actinomycetemcomitans***. Metabolic activity of *C. albicans* yields a purple or orange color upon reduction of MTT and XTT respectively. Incubation of *A. actinomycetemcomitans* does not result in a significant color change.

Supplementary Figure S2 **Complementation of the *luxS*-effect using spent medium of wild-type *A. actinomycetemcomitans***. Addition of 4 and 6 h-old wt-spent medium to mixed species biofilms of *C. albicans* ATCC 10231 and *A. actinomycetemcomitans* results in inhibition of biofilm formation. Addition of spent medium of *A. actinomycetemcomitans luxS* did not affect biofilm formation.

Supplementary Figure S3 **Effect of spent medium of wild-type *A. actinomycetemcomitans* on growth kinetics of *C. albicans* SC5134**. An overnight culture of *C. albicans* was diluted 1:20 or 1:50 in medium consisting of 70% YNB + 30% filter sterilized 4 h-old spent medium of wild-type *A. actinomycetemcomitans*. As a control, the same inoculum was grown in 70% YNB + 30% fresh TSB + YE. No significant differences were observed illustrating the absence of any toxicity related agent in the spent medium of wild-type *A. actinomycetemcomitans*.
