# Supplementary material for: AI-2 of Aggregatibacter actinomycetemcomitans inhibits Candida albicans biofilm formation
Source: Front Cell Infect Microbiol. 2014 Jul 21;4:94. doi: 10.3389/fcimb.2014.00094 (PMC4104835; doi:10.3389/fcimb.2014.00094)

Supplementary Figure S1


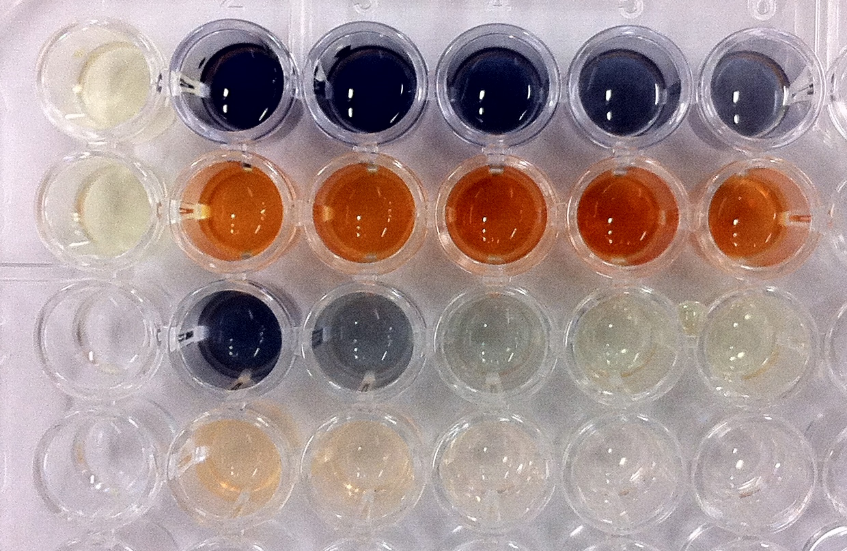


*C. albicans*

*A. actinomycetemcommitans*

Culture density (OD_600_)

- 0.25 0.125 0.0625

MTT

MTT

XTT

XTT


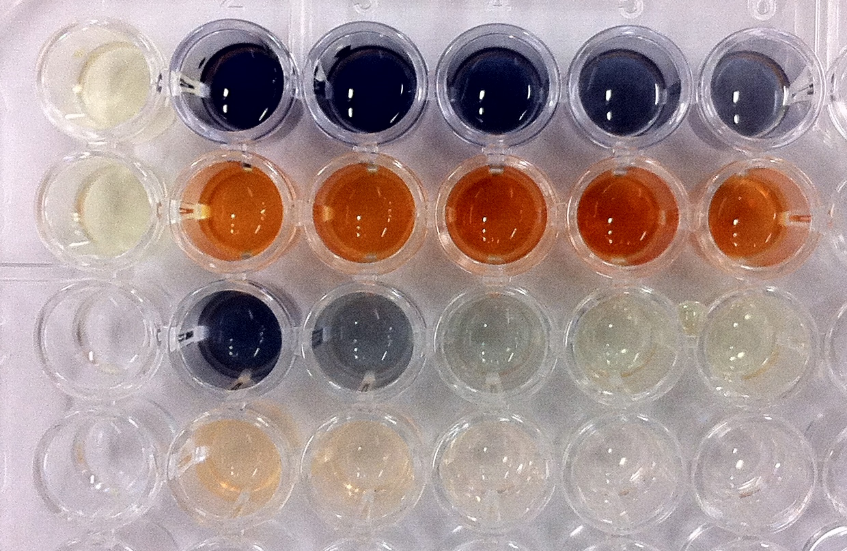


Supplementary Figure S2

Supplementary figure S3.


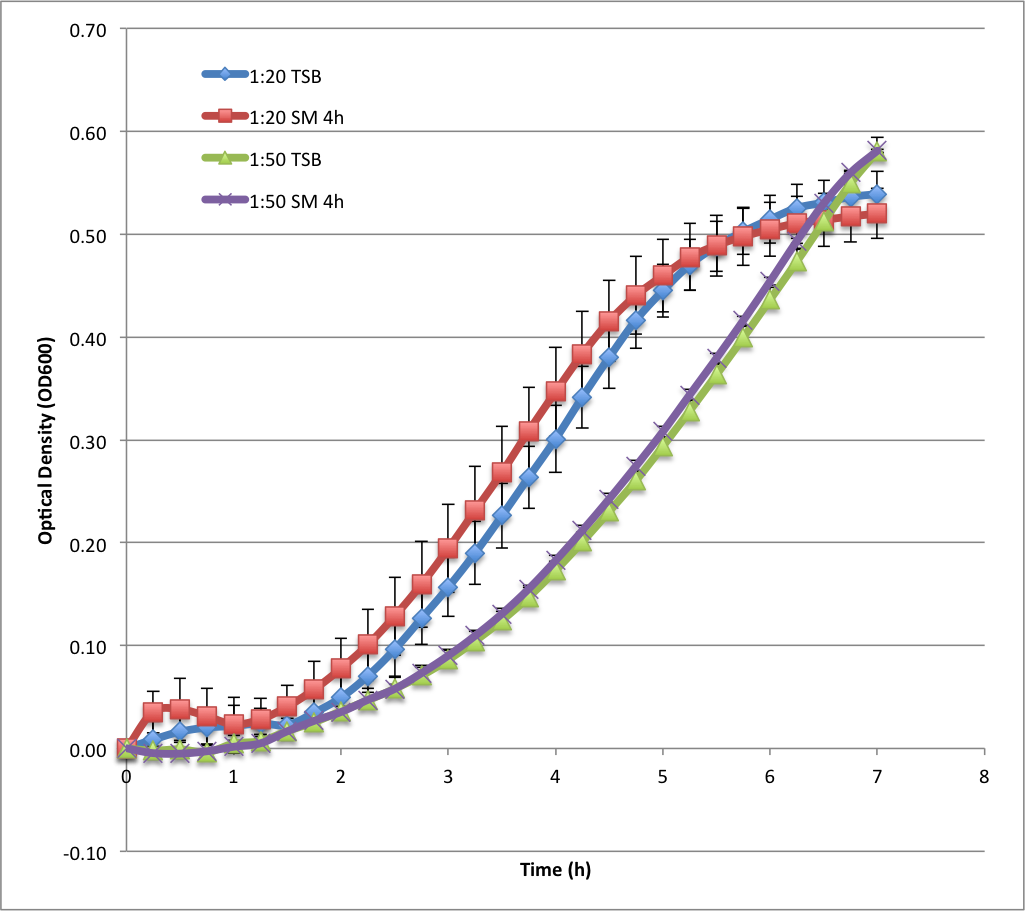

Supplement: Supplementary Figure S1 — Comparison of MTT and XTT reduction by C. albicans SC5314 and A. actinomycetemcomitans. Metabolic activity of C. albicans yields a purple or orange color upon reduction of MTT and XTT respectively. Incubation of A. actinomycetemcomitans does not result in a significant color change. [file DataSheet1.DOCX]
